# Interpregnancy interval and maternal postpartum depression and anxiety: findings from the APrON cohort

**DOI:** 10.1093/eurpub/ckag153

**Published:** 2026-08-19

**Authors:** Peighton Johnson, Nicole Letourneau, Maryam Kebbe

**Affiliations:** Department of Kinesiology, University of New Brunswick, Fredericton, NB, Canada; Department of Nursing, University of Calgary, Calgary, AB, Canada; Department of Kinesiology, University of New Brunswick, Fredericton, NB, Canada

## Abstract

Shorter interpregnancy intervals (IPI) are linked to adverse maternal health outcomes, such as pre-eclampsia and gestational diabetes mellitus. The link between IPI and maternal mental health has not been examined despite its potential clinical implications. The aim of this study was to examine associations between IPI and postpartum symptoms of depression (PPD) and anxiety (PPA). We retrieved data from the APrON study. Mothers (*n = *613) completed the Edinburgh Postpartum Depression Scale to assess depression and the Symptoms Checklist-90 to assess anxiety within 1 year of the second child’s delivery. IPI was categorized into <6, 6*–*11, 12*–*17, 18*–*23, 24*–*59, and 60*–*119 months. Logistic generalized linear modeling was performed. Mothers ≥35 years of age had twice the odds of PPD (OR = 2.06, 95% CI: 1.25–3.36, *P = .*0041) compared to mothers <35 years. Mothers with high social support had 54% lower odds of PPD (OR = 0.46, 95% CI: 0.29*–*0.72, *P = .*00074) compared to mothers with low social support. Mothers with a history of PPD and/or PPA had 3× greater odds of PPD (OR = 3.00, 95% CI: 1.47*–*5.96, *P = .*0020) compared to those without a history. Social support postpartum and history of depression and/or anxiety were also associated with PPA (*P < .*05). IPI was not related to maternal PPD or PPA in this cohort. Comprehensive mental health support programs and resources are warranted not only postnatally, but prenatally and throughout gestation, particularly in older mothers and those with mental illness history.

## Introduction

An interpregnancy interval (IPI) represents the length of time between a previous delivery and subsequent conception. Due to increased health risks with shorter IPIs, the World Health Organization recommends an interval of at least 24 months following a live birth to reduce adverse birth outcomes [1]. IPIs shorter than 18–24 months are associated with higher risks of adverse maternal, perinatal, and infant outcomes [2–4]. Specifically, there are increased risks for preterm delivery, gestational diabetes mellitus, pre-eclampsia, small-for-gestational-age infants, and infant mortality [2–4]. Previous research has also found that among mothers with a previous Cesarean delivery, a short IPI (<6 months) was associated with increased risk of uterine rupture [3]. Inadequate maternal repletion, insufficient time to lose gestational weight gain postpartum, and incomplete healing of the uterine incision post-Cesarean section may explain why a short IPI may increase adverse maternal and infant health outcomes [3].

IPI is routinely addressed in clinical and public health guidelines due to its established associations with adverse obstetric and neonatal outcomes. However, most evidence and recommendations related to IPI focus primarily on physical health outcomes, with considerably less attention given to maternal mental health. A recent systematic review highlighted that no research reports on the association between IPI and maternal postpartum depression (PPD) [3], and to our knowledge, no studies have examined maternal postpartum anxiety (PPA) as an outcome either. Studies have repeatedly demonstrated that more than 1 in 10 mothers experience symptoms of depression, while comparable rates of mothers suffer from anxiety symptoms [5–8]. Further, research conducted among mothers who had given birth in Canada reported that 17.9% had PPD symptom levels above the validated screening cutoff, indicating likely PPD, while 13.8% had PPA symptom levels above the validated screening cutoff, indicating likely PPA [9]. Given the high burden of postpartum mental health disorders and the absence of evidence examining their relationship with IPI, investigating this association may help determine whether birth spacing should be considered as part of strategies to promote maternal and infant well-being.

Several factors may support a link between IPI and PPD and PPA, such as nutritional depletion and caregiving responsibilities that may arise with short IPIs. For example, pregnancy and lactation impose considerable nutritional demands on mothers, necessitating adequate time for replenishment of essential nutrients such as iron, folate, calcium, and omega-3 fatty acids [10]. These nutrients play a crucial role not only in maternal physical health but also in mental well-being. For instance, deficiencies in omega-3 fatty acids and folate have been linked to increased risks for depression and anxiety [11, 12]. Iron deficiency, which is common postpartum, has also been associated with depressive symptoms [13]. Mothers with short IPIs may be at higher risk of nutritional depletion, which in turn could contribute to PPD and PPA. In addition to nutritional depletion, the burden and stress of caring for multiple young children simultaneously can significantly impact maternal mental health. The transition to motherhood is a challenging period that demands considerable physical, emotional, and psychological resources [14]. Short IPIs can lead to overlapping caregiving responsibilities, with mothers managing the needs of an infant and a toddler concurrently. This situation can reduce the time available for self-care and increase stress levels, a well-established predictor of PPD and PPA [15, 16].

The specific role of IPI is clinically and public health relevant for several reasons. First, IPI is a modifiable reproductive factor that can be influenced through contraceptive counseling, family cplanning services, and postpartum care planning. Understanding its relationship with maternal mental health may therefore help inform more holistic postpartum counseling that integrates both physical recovery and psychological well-being. Second, existing clinical guidelines on birth spacing primarily focus on obstetric and neonatal outcomes, with less emphasis on maternal mental health outcomes. Clarifying this relationship may help refine recommendations and support more comprehensive counseling in perinatal care settings. Third, PPD and PPA remain highly prevalent and are associated with substantial long-term consequences for both mothers and child development; identifying potentially modifiable upstream factors such as IPI could improve prevention strategies.

Given the need to further understand if birth spacing is associated with maternal health outcomes, this study aims to examine the association between IPI and postpartum maternal depression and anxiety following the subsequent pregnancy. We hypothesized that shorter IPIs would be associated with increased maternal depression and anxiety symptoms in the postpartum period following the subsequent pregnancy. Understanding the complex interplay between birth spacing and maternal mental health can inform the development of effective interventions aimed at supporting maternal well-being across the reproductive lifespan.

## Methods

### Study population

Data for this study were obtained from the ongoing Alberta Pregnancy Outcomes and Nutrition (APrON) longitudinal cohort study that was established in 2009 [17]. APrON includes data on maternal and infant nutrition, mental health, infant feeding practices, and infant/child health and development. All participants were recruited from APrON. Participants were eligible if they were less than 27 weeks pregnant at the time of enrollment in the APrON study, were 16 years of age or older, and were fluent in both written and spoken English. More detail on recruitment and participation rates can be found in the parent study [17].

Our sample for this secondary data analysis included mothers with two consecutive singleton births, who met the above inclusion criteria. Mothers who had delivered multiples, had more than two children enrolled in APrON, and/or had missing information for any variable of interest, were excluded. Additionally, mothers whose first pregnancy ended in a spontaneous or induced abortion at less than 20 weeks’ gestation, stillbirth, or neonatal death were excluded, as guidelines for pregnancy spacing differ for pregnancies following a loss.

### Data collection

#### Exposure variables

IPI was explored as a categorical variable in accordance with existing recommendations and previous literature [1, 18, 19]. Namely, IPI was grouped into the following categories: <6 months, 6*–*11 months, 12*–*17 months, 18*–*23 months (reference), 24*–*59 months, and 60*–*119 months [1, 18, 19].

#### Outcome variables

Mothers completed the validated Edinburgh Postnatal Depression Scale (EPDS) and Symptoms Checklist-90 (SCL-90) at 3, 6, and 12 months following their first child’s birth. Total depression and anxiety scores were summed for each time point. For the current analyses, we defined the postpartum exposure window as 0–12 months following the birth of the second child. Within this window, if multiple assessments were available, we selected the assessment with the highest recorded depression and anxiety scores for each participant to capture peak symptom burden during the postpartum period.

The EPDS is a validated 10-item questionnaire. EPDS scores of 10 or higher indicate depression; therefore, participants were categorized as having depression (≥10) or not having depression (<10) [20]. The SCL-90 includes a subscale of 10 questions (#1, 5, 7, 10, 13, 16, 19, 21, 23, 25) that pertain directly to anxiety. These questions were summed to determine scores for anxiety, which were categorized into cutoff points of ≥75th quartile as having anxiety or <75th quartile as not having anxiety. This approach is consistent with other epidemiological literature [21].

Potential covariates measured at the data collection visit of the first pregnancy included maternal age, race, parity, income, and smoking. Social support was measured twice during the first pregnancy, and at 3 and 12 months following the first pregnancy. The highest recorded social support score was used for each interval. Covariates were categorized as follows: age (≥35 years of age or <35 years of age), race (Caucasian or non-Caucasian), parity (0 or ≥1 live births prior to enrollment), income (≥$70 000 CAD or <$70 000 CAD), smoking (yes or no), social support during the first pregnancy (≥75th percentile as having high support or <75th percentile as having low support), and social support following the first pregnancy (≥75th percentile as having high support or <75th percentile as having low support). These variables were theoretically judged to be important with respect to mental health outcomes, as supported by previous literature [22–26]. All covariates were obtained through self-report and researcher-developed questionnaires except for social support. Social support was assessed via the Social Support Questionnaire (SSQ) [27]. Scores on the SSQ could range from 0 to 16, with higher scores indicating more social support.

### Statistical analysis

Sociodemographic characteristics of the cohort were summarized using frequencies and measures of central tendencies. A binary logistic generalized linear regression model was used to determine the relationship between IPI (categorical) and depression and anxiety (binary). Both raw and adjusted models were examined. Using the theoretically chosen covariates, we employed a stepwise approach to finalize covariates for our analysis. Initially, univariate analyses were conducted to evaluate the relationship between each covariate and the outcomes of interest (depression and anxiety symptoms). Covariates with a significance level of *P* ≤ .25 were advanced to the subsequent step [28]. To determine the optimal model, covariates yielding the strongest fit according to the Akaike Information Criterion (AIC) were chosen. These included maternal age, income, social support following the first pregnancy, and history of depression and/or anxiety for both PPD and PPA models. Analyses were performed in R 4.1.1 with *P* < .05 considered significant.

## Results

Of the original cohort (*n = *2189), 613 participants (28.0%) were included in the analytic sample with complete data on IPI and all covariates of interest. Comparisons of available baseline characteristics indicated no meaningful differences between included and excluded participants (*P* > .05). In this sample, the included 613 mothers were 32.4 ± 3.7 years on average. The majority were Caucasian (88.3%), had an annual income ≥$70 000 CAD (82.7%), completed post-secondary education (93.0%), were married (91.8%), and had one or more children prior to enrolling in this study (69.3%). Mothers had a similar average social support score during and after the first pregnancy (14.6 ± 2.4 and 14.8 ± 2.2, respectively).

**Figure 1:**
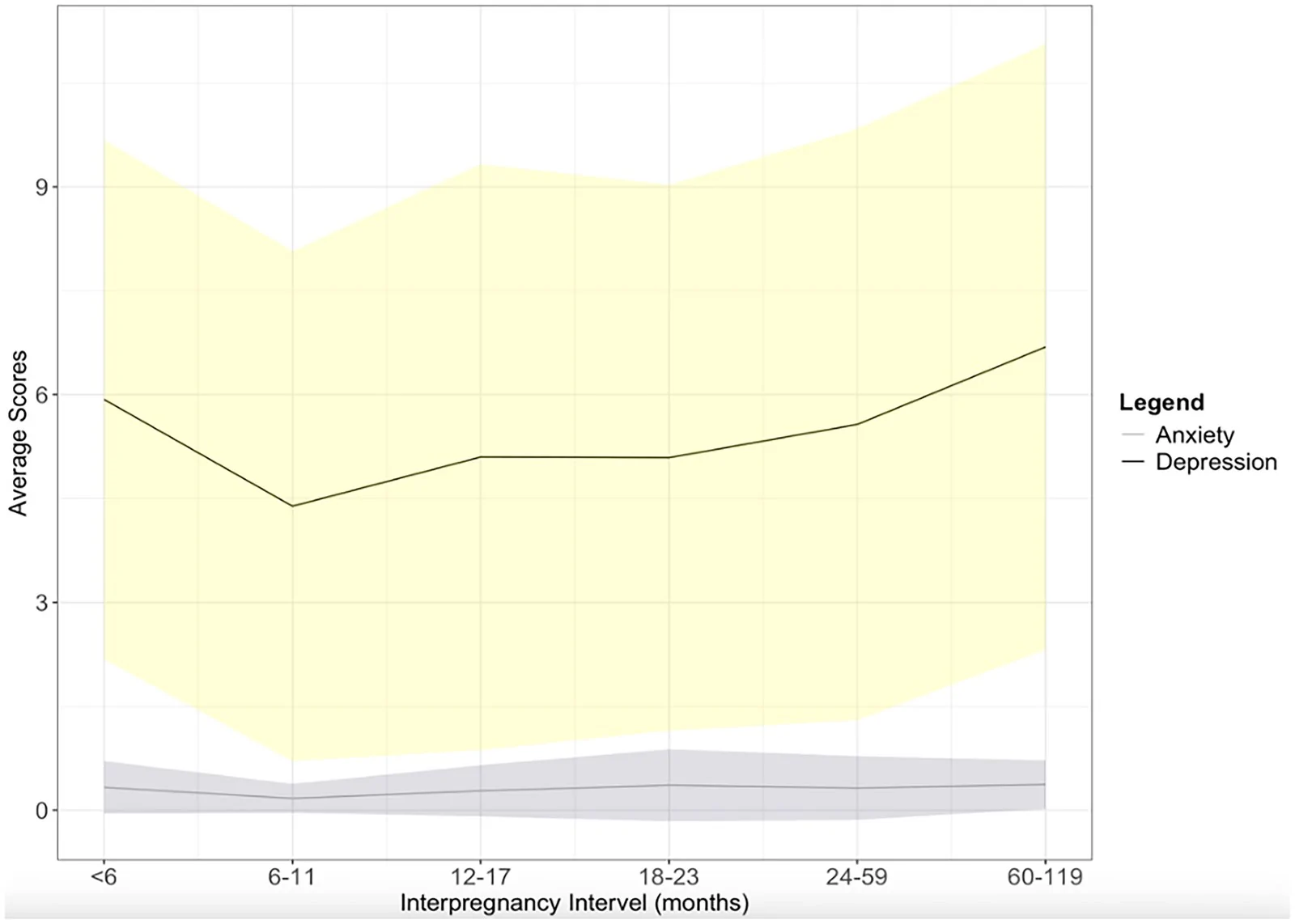
Average depression scores from the Edinburgh Postpartum Depression Scale (EDPS) and Symptoms Checklist-90 (SCL-90) anxiety scores categorized across interpregnancy intervals. Participants’ individual scores were summed, and all scores were averaged across respective IPI categories. Interpregnancy intervals were categorized based on World Health Organization recommendations.

Across interpregnancy interval categories, there were 15 participants in the <6 months group, 91 in the 6–11 months group, 166 in the 12–17 months group, 123 in the 18–23 months (reference) group, 186 in the 24–59 months group, and 32 in the 60–119 months group. Approximately 16.0% participants met criteria for PPD and PPA. Mothers had an average depression score of 5.24 ± 4.12 and an average anxiety score of 0.31 ± 0.43. Most did not have a history of PPD (96.1%) or depression and anxiety diagnoses (92.8%). Depression and anxiety scores were not significantly different across IPI durations at the time of outcome assessment (Fig. 1). Demographic information and characteristics of the cohort are reported in Table 1.

**Table 1:** Characteristics of the study population.

Characteristics	Mean ± SD *N* (%)
Average maternal age (years)	32.42 ± 3.72
Average depression score	5.24 ± 4.12
Average anxiety score	0.31 ± 0.43
Average social support score during first pregnancy	14.63 ± 2.38
Average social support score following first pregnancy	14.79 ± 2.17
Race	
Caucasian	541 (88.25)
Non-Caucasian	72 (11.75)
Socioeconomic status^a^	
≥$70 000 CAD	507 (82.71)
<$70 000 CAD	106 (17.29)
Marital status	
Married	563 (91.84)
Not married	50 (8.16)
Education	
Completed post-secondary	570 (92.99)
Less than post-secondary	43 (7.01)
Parity^b^	
0	188 (30.67)
≥1	425 (69.33)
History of previous postpartum depression	
Yes	24 (3.92)
No	589 (96.1)
History of depression and/or anxiety	
Yes	44 (7.18)
No	569 (92.82)

a Socioeconomic status is reported based on annual income in Canadian Dollars (CAD).

b Parity indicates number of live births the participant delivered prior to enrollment.

### Relationship between IPI and postpartum depression

In the unadjusted model for depression, mothers with an IPI of 60*–*119 months had significantly higher odds of PPD compared to the recommended 18–23 months (2.70, 95% CI: 1.11–6.42, *P* = .03). This relationship was no longer significant when adjusted for covariates (Table 2). In regards to covariates, compared to mothers <35 years of age, mothers who were ≥35 years of age had twice the odds of PPD (2.06, 95% CI: 1.25–3.36, *P* = .0041). Further, mothers with a history of PPD and/or PPA had 3× greater odds of PPD compared to those without a history (3.00, 95% CI: 1.47–5.96, *P* = .0020). Lastly, in mothers with high social support, the odds of PPD were 54% lower than women with low social support (0.46, 95% CI: 0.29–0.72, *P* = .00074).

**Table 2:** Interpregnancy interval and depression results (raw and adjusted model data).^a^

IPI	Odds ratio	95% CI
Raw model		
<6 months	1.87	0.48–6.11
6–11 months	0.50	0.20–1.16
12–17 months	0.98	0.52–1.86
18–23 months	–	–
24–59 months	1.13	0.62–2.10
60–119 months	2.70	1.11–6.42
Adjusted model		
<6 months	1.30	0.31–4.71
6–11 months	0.51	0.19–1.21
12–17 months	1.12	0.58–2.18
18–23 months	–	–
24–59 months	0.98	0.52–1.87
60–119 months	1.72	0.66–4.37
Covariates		
Age	2.06	1.25–3.36
Income	0.70	0.39–1.28
History of depression and/or anxiety	3.00	1.47–5.96
Social support following first pregnancy	0.46	0.29–0.72

a The Edinburgh Postpartum Depression Scale (EPDS) was used to measure postpartum depression, with higher scores (≥10) indicating postpartum depression. EPDS was filled out within 12 months of the delivery date of the second child enrolled in the study. A binary logistic regression model was employed. IPI of 18*–*23 months was used as the reference. The Edinburgh Postpartum Depression Scale (EPDS) was used to measure postpartum depression, with higher scores (≥10) indicating postpartum depression. EPDS was filled out within 12 months of the delivery date of the second child enrolled in the study. A binary logistic regression model was employed. IPI of 18*–*23 months was used as the reference. Covariates in the adjusted model included age [<35 years of age (reference) or ≥35 years of age], annual income [<$70 000 (reference) or ≥$70 000 CAD), social support [low (reference) or high], and history of depression and/or anxiety [no (reference) or yes]. “Reference” denotes the comparator group against which other groups are compared.

CI, confidence interval.

### Relationship between IPI and postpartum anxiety

No significant differences (*P > .*05) were found between IPI and PPA in the unadjusted and adjusted models (Table 3). In regards to covariates, mothers with a history of PPD and/or PPA had 2.5× greater odds of PPA compared to those without a history (2.46, 95% CI: 0.99–6.01, *P = .*048). Further, in mothers with high social support, the odds of PPA were 54% lower than women with low social support (0.46, 95% CI: 0.28–0.75, *P = .*0020).

**Table 3:** Interpregnancy interval and anxiety results (raw and adjusted model data).^a^

IPI	Odds ratio	95% CI
Raw model		
<6 months	1.29	0.17–7.11
6–11 months	0.41	0.11–1.22
12–17 months	1.09	0.53–2.22
18–23 months	–	–
24–59 months	1.10	0.59–2.07
60–119 months	1.37	0.49–3.66
Adjusted model		
<6 months	0.91	0.11–5.60
6–11 months	0.37	0.095–1.14
12–17 months	1.06	0.50–2.24
18–23 months	–	–
24–59 months	1.05	0.55–2.03
60–119 months	0.97	0.32–2.74
Covariates		
Age	1.46	0.85–2.49
Income	0.66	0.34–1.33
History of depression and/or anxiety	2.46	0.99–6.01
Social support following first pregnancy	0.46	0.28–0.75

a The anxiety scale of the Symptoms-Checklist-90 (SCL-90) was used to measure anxiety symptoms. SCL-90 was filled out within 12 months of the delivery date of the second child enrolled in the study. A binary logistic regression model was employed. IPI of 18*–*23 months was used as the reference. Covariates in the adjusted model included age [<35 years of age (reference) or ≥35 years of age], annual income [<$70 000 (reference) or ≥$70 000 CAD], social support [low (reference) or high], and history of depression and/or anxiety [no (reference) or yes]. “Reference” denotes the comparator group against which other groups are compared.

CI, confidence interval.

## Discussion

Unadjusted results showed a significant association between longer IPI and maternal symptoms of depression; however, this association was significantly attenuated when the model was adjusted for covariates suggesting that IPI is not related to maternal symptoms of depression in this cohort. Maternal age was positively associated with the odds of developing high symptoms of PPD, while social support postpartum and a history of depression and/or anxiety were inversely and positively, respectively, associated with PPD and PPA. Our study also found that mothers with high social support postpartum had significantly lower odds of PPD compared to those with low social support, reducing the likelihood by over half. Additionally, we found social support to be inversely related to PPA, reducing the likelihood by over half, and that history of depression and/or anxiety increased the likelihood by almost 2.5×. These insights emphasize the multifaceted nature of postpartum mental health and the importance of a holistic approach in both research and clinical practice.

Our findings suggest that in this cohort, IPI was not associated with maternal symptoms of PPD or PPA. However, we found that mothers 35 years of age and older have more than a 100% increase in the odds of developing PPD, compared to mothers younger than 35 years of age. This finding is consistent with other research which shows higher rates of PPD among older mothers (>35 years of age) compared to younger mothers [22, 29]. Higher odds of developing PPD for older mothers may be linked to a variety of factors, including a reduced sense of peer support and societal expectations on maternal childbearing age [22, 30]. In addition, studies have suggested that multiple births, greater obstetrical difficulties, heightened use of reproductive technologies, and a history of depression may play a role in increased PPD risk [22, 29, 31], the latter being observed in our study. In contrast, our findings are not consistent with studies that have demonstrated an inverse relationship between maternal age and PPD [22, 31, 32]. These studies suggest that the expected higher socioeconomic status, relationship stability, and presumed maturity of older mothers may help protect against depression, compared to circumstances of younger mothers [22, 31, 32]. Although our cohort consisted primarily of married women from high socioeconomic status, there may have been alternative factors, such as genetic predispositions, that were not adjusted for and that influenced our findings.

Research consistently demonstrates the significant impact of social support on PPD among new mothers. For instance, Gheorghe *et al.* [9] found that mothers who reported adequate social support were less likely to develop symptoms of PPD and PPA. This relationship can be attributed to various factors: social support buffers against stressors associated with caregiving and adjustment to motherhood, provides emotional reassurance, and may enhance coping strategies [33]. Moreover, the absence of social support has been linked to increased feelings of isolation and being overwhelmed, which are known risk factors for PPD and PPA [34].

Our study found no significant odds of developing PPA across IPIs, suggesting that other factors may predict the risk of developing PPA. Indeed, we found social support to be inversely related to PPA, reducing the likelihood by over half, and that history of depression and/or anxiety increased the likelihood almost 2.5×. History of anxiety/depression/mood disorders, including trait anxiety and postpartum state anxiety, have similarly previously been shown to be significant predictors of PPA [9, 35]. Specifically, Gheorghe *et al.* [9] found that mothers in Canada who have a history of depression were 3.4× more likely to develop symptoms of PPA compared to those who did not have a history of depression. In addition to mental health, physical health is another potential indicator of PPA. Mothers in poor, fair, or good physical health were 2.0× more likely to develop symptoms associated with PPA, compared to those who reported very good or excellent physical health [9]. Together, this literature suggests that mothers’ previous mental and physical health conditions play a large role in the development of PPA.

Screening for depression and anxiety symptoms throughout gestation and in the postpartum period is important to identify symptoms of maternal PPD and PPA. Fostering supportive healthcare networks throughout gestation and for new mothers should be prioritized, as support systems can enhance maternal well-being and contribute to healthier outcomes for both mothers and their infants. Future research should explore specific types and sources of support that are most beneficial in mitigating postpartum mental health challenges, thereby informing targeted interventions to optimize maternal mental health during this vulnerable period.

Strengths of this study include the use of a large Canadian cohort with information directly pertaining to the postpartum period. Using commonly used and validated tools for depression (EPDS) and anxiety (SCL-90) symptoms throughout the postpartum period enhanced the validity of derived depression and anxiety scores. However, our cohort was not diverse, as it primarily consisted of Caucasian, married mothers. Our cohort was limited to mothers with a high socioeconomic status, which may impact their ability to provide childcare, nutritious meals, and a variety of other factors, thereby impacting their stress and anxiety levels and overall mental health. In addition, although this study was adequately powered to detect moderate associations between interpregnancy interval and PPD and PPA in the larger IPI categories, it had more limited power to detect small effect sizes and associations within the least common IPI categories (<6 months and 60–119 months). Therefore, while our findings do not support a clinically meaningful association between IPI and postpartum mental health outcomes, smaller associations cannot be excluded.

Maternal mental health is important for the well-being of the mother and the infant. Therefore, screening mothers for PPD and PAA is critical to identify any symptoms, and thereafter intervening to reduce the number of and degree to which mothers are suffering from negative postpartum mental health implications. Proactive mental health support programs and resources may be warranted not only prenatally and throughout gestation, but also postnatally, particularly in older mothers, mothers with low levels of social support, and mothers with a history of mental illness.

## Data Availability

The data underlying this article may be shared on reasonable request to the corresponding author. Key points IPI is not directly related to maternal postpartum depression and anxiety. Maternal age, social support, and mental health history are related to postpartum depression and anxiety. Perinatal mental health support programs are needed, especially for older mothers with a mental illness history.
